# A continuum of genetic mixing for conservation management along the (mal)adaptation spectrum: A comment on Hoffmann et al.

**DOI:** 10.1111/eva.13196

**Published:** 2021-01-29

**Authors:** Erika Crispo, Alison M. Derry, Steven P. Brady

**Affiliations:** ^1^ Department of Biology Pace University New York NY USA; ^2^ Département des Sciences Biologiques Université du Québec à Montréal Montréal QC Canada; ^3^ Biology Department Southern Connecticut State University New Haven CT USA

**Keywords:** adaptation, conservation, evolutionary rescue, genetic rescue, genetic variation, maladaptation

## Abstract

When restoring gene flow for conservation management, genetic variation should be viewed along a continuum of genetic divergence between donor and recipient populations. On the one hand, maintaining local adaptation (low divergence between donors and recipients) can enhance conservation success in the short term. On the other hand, reducing local adaptation in the short term by increasing genetic diversity (high divergence between some donors and recipients) might have better long‐term success in the face of changing environmental conditions. Both Hoffman et al. (2020) and a paper we previously published in a Special Issue on Maladaptation in Applied Conservation (Derry et al., 2019) provide frameworks and syntheses for how best to apply conservation strategies in light of genetic variation and adaptation. A key difference between these two studies was that whereas Derry et al. (2019) performed a quantitative meta‐analysis, Hoffman et al. (2020) relied on case studies and theoretical considerations, yielding slightly different conclusions. We here provide a summary of the two studies and contrast of the main similarities and differences between them, while highlighting terminology used to describe and explain main concepts.

We were encouraged to see Hoffmann et al. (2020) provide a clarifying treatment of the numerous genetic‐based strategies used in conservation (e.g. genetic recue, evolutionary rescue, genetic provenancing and hybridization). The authors contend that there is no silver bullet technique to restore populations. Rather, the choice of strategy should consider how its implementation will affect genetic variation, which is best viewed along a continuum of divergence between donor and recipient populations. We could not agree more, and indeed advocated similar tack one year earlier when we published a conceptual framework and synthesis on the topic (Derry et al., 2019). We believe the two papers provide complementary insights that will best advance the field by contrasting their main differences here.

Both papers framed strategies in terms of meeting short‐term versus long‐term adaptation targets of genetic variation, which we referred to as managing for ‘adaptive state’ versus ‘adaptive process’. A key difference, however, was our use of quantitative meta‐analysis to evaluate effectiveness of different conservation strategies; Hoffmann et al. (2020) relied on case‐study narratives. Both papers define and contrast different conservation strategies used for population management, which Hoffmann et al. (2020) call different ‘genetic mixing strategies’ (table 1 in Hoffmann et al., 2020) and which we placed on a gradient of adaptive divergence between recipient and donor populations (figure 1b in Derry et al., 2019). Our gradient spans from strategies that have traditionally ignored genetic variation to those that leverage it for success; Hoffmann et al. (2020) focused only on the latter. We here present a combined list of strategies to provide a bridge of continuity of terms and concepts between the two papers (Table 1).

**TABLE 1:** Conservation strategies described and defined in Derry et al. (2019) and Hoffmann et al. (2020), ordered along a gradient of genetic mixing from low (conserving adaptive state) to high (conserving adaptive process)

Conservation strategy	Defined in Derry et al. (2019)	Defined in Hoffmann et al. (2020)	Definition	State or process	Note
Assisted migration	Yes	No	Moving individuals to a more suitable location	State	Matches phenotype and environment
Demographic rescue	Yes	No	Stocking individuals to increase population size	State	Traditionally ignores genetic diversity
Genetic rescue	Yes	Yes	Masking deleterious recessive alleles and reducing inbreeding	State	Can increase evolutionary potential by increasing genetic diversity
Genotype provenancing	No	Yes	Matching phenotypes and environments so that populations are at their phenotypic optima	State or process (depends on whether choosing to restore population with genotypes/ phenotypes similar to or different from those already present)	Shares similarities with assisted migration and demographic rescue
Transgenerational plasticity/acclimatization	Yes	No	Inducing heritable phenotypes in laboratory/hatchery setting	Process	Creates variation not previously encountered
Evolutionary rescue	Yes	Yes	Increasing genetic variation upon which selection can act	Process	Increases the deviation in phenotypes from the optimum in the population; focusses on long‐term potential
Interspecific hybridization	Yes	Yes	Creating novel genotypes by crossing species	Process	Can lead to breakdown of coadapted gene complexes; success might vary with generation (F1, F2, F3…)
Genomic selection	Not specifically, but we discuss molecular tools in Box 1 of Derry et al. (2019)	Yes	Using genomic tools to identify high fitness genotypes	Process	Considered a process because it alters genetic make‐up of a population but does not focus on long‐term change
Allele modification/genetic engineering	Yes (genetic engineering in Box 2 of Derry et al. 2019)	Yes (allele modification)	Using biotechnology to directly alter genetic make‐up of individuals	Process	Creates novel genetic variation but does not increase genetic diversity in a population
Species replacement	No	Yes	Replacing one species with a functionally similar one in the community	Neither	Maintains the state of the ecosystem but does not conserve individual species

One contrast between conclusions is that we found that genetic and evolutionary rescue tended to increase fitness across multiple generations relative to immediately after conservation intervention (figure 2 in Derry et al., 2019). Hoffmann et al. (2020) claim that fitness benefits of these strategies were more likely to occur in the F1 and F2 generations than in later generations, but that this effect should depend on the history of recipient population size (table 2 in Hoffmann et al., 2020). This discrepancy highlights how different methodologies (quantitative analysis vs. case study and theoretical approaches) can yield different conclusions, and the importance of considering nuances of individual systems.

Importantly, Hoffmann et al. (2020) point out that the value of increasing genetic diversity in the recipient population should increase with both the degree of environmental change and the size of the recipient/target populations (figure 4 in Hoffmann et al., 2020). We expect, though, that the importance of genetic diversity in relation to population size and environmental change would not be linear, as depicted in figure 4 of Hoffmann et al. (2020), and instead, the slope would be steepest at the smallest population sizes. The reasoning is that genetic drift plays a larger role in small populations (Lande, 1988; Nei & Tajima, 1981) whereas selection is more efficient in large populations (Ellstrand & Elam, 1993; Falconer & MacKay, 1996; Frankham et al., 2010; Lande, 1988; Willi et al., 2006); further empirical research, however, is needed on small and isolated populations to understand their adaptive potential in response to selection (Hoffmann et al., 2017; Wood et al., 2016).

In conclusion, we hope that the frameworks of genetic mixing along a gradient generated in Derry et al. (2019) and Hoffmann et al. (2020) will be considered and implemented in many empirical systems in relation to conservation scenarios. An evolutionary focus in conservation management will be increasingly important as human‐induced changes to our natural world continue to expand at a rapid rate.

## CONFLICT OF INTEREST

There is no conflicts of interest to declare.

## References

[eva13196-bib-0001] Derry, A. M. , Fraser, D. J. , Brady, S. P. , Astorg, L. , Lawrence, E. R. , Martin, G. K. , Matte, J. M. , Negrín Dastis, J. O. , Paccard, A. , Barrett, R. , Chapman, L. J. , Lane, J. E. , Ballas, C. G. , Close, M. , & Crispo, E. (2019). Conservation through the lens of (mal)adaptation: Concepts and meta‐analysis. Evolutionary Applications, 12(7), 1287–1304. 10.1111/eva.12791 31417615 PMC6691223

[eva13196-bib-0002] Ellstrand, N. C. , & Elam, D. R. (1993). Population genetic consequences of small population size: Implications for plant conservation. Annual Review of Ecology and Systematics, 24, 217–242. 10.1146/annurev.es.24.110193.001245

[eva13196-bib-0003] Falconer, D. S. , & Mackay, T. F. C. (1996). Introduction to quantitative genetics (4th ed.). Longman.

[eva13196-bib-0004] Frankham, R. , Ballou, J. D. , & Briscoe, D. A. (2010). Introduction to conservation genetics. Cambridge University Press.

[eva13196-bib-0005] Hoffmann, A. A. , Miller, A. D. , & Weeks, A. R. (2020). Genetic mixing for population management: From genetic rescue to provenancing. Evolutionary Applications, 14(3), 1–19. 10.1111/eva.13154 PMC7980264 33767740

[eva13196-bib-0006] Hoffmann, A. A. , Sgrò, C. M. , & Kristensen, T. N. (2017). Revisiting adaptive potential, population size, and conservation. Trends in Ecology & Evolution, 32(7), 506–517. 10.1016/j.tree.2017.03.012 28476215

[eva13196-bib-0007] Lande, R. (1988). Genetics and demography in biological conservation. Science, 241(4872), 1455–1460. 10.1126/science.3420403 3420403

[eva13196-bib-0008] Nei, M. , & Tajima, F. (1981). Genetic drift and estimation of effective population size. Genetics, 98(3), 625–640. 17249104 10.1093/genetics/98.3.625 PMC1214463

[eva13196-bib-0009] Willi, Y. , Van Buskirk, J. , & Hoffman, A. A. (2006). Limits to the adaptive potential of small populations. Annual Review of Ecology, Evolution, and Systematics, 37, 433–458. 10.1146/annurev.ecolsys.37.091305.110145

[eva13196-bib-0010] Wood, J. L. A. , Yates, M. C. , & Fraser, D. J. (2016). Are heritability and selection related to population size in nature? Meta‐analysis and conservation implications. Evolutionary Applications, 9, 640–657. 10.1111/eva.12375 27247616 PMC4869407

